# Editorial: HIV and Viral Co-infections

**DOI:** 10.3389/fmicb.2021.731337

**Published:** 2021-08-31

**Authors:** Carlos Brites, Álvaro H. Borges, Eduardo Sprinz, Kimberly Page

**Affiliations:** ^1^Federal University of Bahia, Salvador, Brazil; ^2^Statens Serum Institut (SSI), Copenhagen, Denmark; ^3^Universidade Federal do Rio Grande do SUL, Porto Alegre, Brazil; ^4^University of New Mexico, Albuquerque, NM, United States

**Keywords:** HIV, viral infections, hepatitis, cancer, HTLV-1

Viral coinfections are common findings in HIV infected patients, notably those who share a common route of infection with HIV (da Silva Neto et al., 2020). Some of those coinfections can be silent and do not cause significant impact on HIV disease nor are affected by it. In contrast, HIV co-infection with certain viral infections can affect the natural history of HIV infection and vice-versa. Hepatitis C virus (HCV) hepatitis B virus (HBV), human T-cell lymphotrophic virus-1 (HTLV-1), and others including human herpes virus-8 (HHV-8) and human papilloma virus (HPV) are among the infectious agents that can modify both their own natural history and the course of HIV infection and disease, when co-infecting the same host.

One striking example relates to the synergistically deleterious interaction of HIV with pro-oncogenic viruses. Viral infection-related cancers such as lymphomas (EBV-related), Kaposi-sarcoma (KSV-related), anal/cervical cancer (HPV-related), and liver cancer (HCV- and/or HBV/HDV-related) have a much higher incidence among HIV+ persons than in the general population (da Silva Neto et al., 2020). Of interest, viral suppression and immune recovery by antiretroviral therapy can largely reduce but not abolish this excess cancer risk (Borges et al., 2016).

Another important example relates to HIV and viral hepatitis viruses co-infection, in which a higher risk for both progression to AIDS and progression to end stage liver disease is observed (Soares et al., 2014; Mocroft et al., 2020). Although there are many published articles on co-infections such as HIV-HCV and HIV-HBV, many aspects of the interaction between HIV and other simultaneous viral infections remain obscure.

The call for papers on this topic aimed to provide new insights on chronic viral infections in patients living with HIV, their characteristics, the interactions between the coinfecting agents, the effect of coinfections on natural history of each virus, and the host immune response. In addition, new epidemiological data on such infections were also of interest, as well as innovative strategies for improvement in the management of affected patients. The current edition of Frontiers in Immunology partially addresses this publication gap featuring several papers focusing on HIV and viral co-infections.

Five articles highlight different aspects of HIV and coinfection HTLV-1 and or HCV: two included molecular epidemiology analyses, one described the cytokines production in HTLV-HCV coinfection, one described the expression of endogenous retrovirus according to HIV subtype, and the last one was focused on factors affecting the linkage to care of HCV infected patients. Regarding their geographical origin, two papers were written by Brazilian authors, 2 came from USA-based researchers, and one from Chinese authors.

Two studies used molecular epidemiological approaches to examine infections in different regions of Brazil. Alencar et al. evaluated the prevalence of HTLV and HIV-1 coinfection using molecular sequencing of samples from patients attending an HIV reference center in Pará state, which is located in the Amazon basin in Brazil (Alencar et al.). They detected a coinfection rate of 1.4% and The results highlight some notable shifts from previously documented epidemiology of HIV and HTLV coinfection, including a much reduced prevalence of co-infection of 1.4%, down from a previously documented 8%, and an absolute predominance of HTLV-1a subtype (Cosmopolitan), subgroup A (Transcontinental), and no detection of HTLV-2 which was much more prevalent historically. The second paper, by Silva et al. detected a high prevalence (17.6%) of HIV-1 infection in a representative sample of men who have sex with men (MSO) in Goiania, capital of Goiás state, Central-West region of Brazil (Silva et al.). They found a predominance of subtype B (67‰, but also presence of F1 (20.5‰, C (3.8%), and BF1 (10.3%). In addition, they found a high (17.3%) prevalence of transmitted drug resistance. The findings of both papers are important contributions for understanding the molecular epidemiology of HIV-1 and HTLV-1 infections in two regions of Brazil outside of well-characterized metropolitan epicenters.

Two papers focused on HCV infection. The first one evaluated the inflammatory cytokines and IL-10 network in patients coinfected by HCV and HTLV (Pereira et al.). The authors detected a trend for a higher production of IFN-γ by coinfected patients, compared with HCV mono-infected ones. They also found a positive correlation between IL-8 and IFN-γ and the regulatory cytokine IL-10. These findings confirmed the capacity of HTLV-1 in modulating the immune response even in patients coinfected with HCV and HIV-1 (Abrahão et al., 2012; Brites et al., 2018). The second paper on HCV (Im et al.) described the characteristics and factors associated with linkage to care of patients diagnosed with chronic hepatitis C in an urban hospital in Chicago, Illinois in the U.S (Im et al.). Although inpatients were more likely to be diagnosed with HCV, outpatients with HCV had almost 5-fold higher odds of being linked to care (OR = 4.9), and of treatment (OR = 4.6), independent of race/ethnicity, sex, and insurance type. Authors elaborate on missed opportunities to provide integrated care and improved health outcomes for HCV infected patients, especially in the in-patient setting.

Finally, the fifth paper presents the results of a study in China (by Li et al.) that evaluated the changes in human endogenous retroviruses (HERV) transcriptional levels according to diverse HIV-1 subtypes in human T cell lines. They observed that the transcriptional levels of HERV were significantly increased by HIV-1 B subtype (HERV-K gag region) and by CRF01_AE and CRF07_BC subtypes (HERV-K pol region). They concluded that different HIV-1 subtypes have different effects on HERV-K transcription levels, and that such effect may be caused by many factors.

Taken together, these 5 articles make a varied panel on different viruses that often coinfect HIV-1 infected patients. They provide new insights on each of listed viruses and contribute to a better understanding of how such agents interact when simultaneously infecting the same individual. Some of the findings also informed on health interventions that can improve the clinical care of patients co-infected with HIV and other viruses. These contributions shed some light on the role of common viral coinfections in HIV.

## Author Contributions

CB edited and formatted the final version of the editorial. All authors contributed with the concept and writing of the manuscript.

## Conflict of Interest

The authors declare that the research was conducted in the absence of any commercial or financial relationships that could be construed as a potential conflict of interest.

## Publisher's Note

All claims expressed in this article are solely those of the authors and do not necessarily represent those of their affiliated organizations, or those of the publisher, the editors and the reviewers. Any product that may be evaluated in this article, or claim that may be made by its manufacturer, is not guaranteed or endorsed by the publisher.

## References

[B1] Abrahão M. H. Lima R. G. Netto E. Brites C. (2012). Short communication: human lymphotropic virus type 1 coinfection modulates the synthesis of cytokines by peripheral blood mononuclear cells from HIV type 1-infected individuals. AIDS Res. Hum. Retroviruses 28, 806–808. 10.1089/aid.2011.0192 22050450

[B3] Borges Á. H. Neuhaus J. Babiker A. G. Henry K. Jain M. K. Palfreeman A.. (2016). Immediate antiretroviral therapy reduces risk of infection-related cancer during early HIV infection. Clin. Infect. Dis. 63, 1668–1676. 10.1093/cid/ciw621 27609756 PMC5146718

[B4] Brites C. Abrahão M. Bozza P. Netto E. M. Lyra A. Bahia F. (2018). Infection by HTLV-1 Is Associated With High Levels of Proinflammatory Cytokines in HIV-HCV-Coinfected Patients. J. Acquir. Immune Defic. Syndr. 77, 230–234. 10.1097/QAI.0000000000001576 29084047

[B5] da Silva Neto M. M. Brites C. Borges Á. H. (2020). Cancer during HIV infection. APMIS 128, 121–128. 10.1111/apm.13020 31990100

[B6] Mocroft A. Lundgren J. Gerstoft J.. (2020). Clinical outcomes in persons coinfected with human immunodeficiency virus and hepatitis C virus: impact of hepatitis C Virus treatment. Clin. Infect. Dis. 70, 2131–2140. 10.1093/cid/ciz601 31504296

[B7] Soares C. C. Georg I. Lampe E. Lewis L. Morgado M. G. Nicol A. F.. (2014). HIV-1, HBV, HCV, HTLV, HPV-16/18, and Treponema pallidum infections in a sample of Brazilian men who have sex with men. PLoS ONE 9:e102676. 10.1371/journal.pone.0102676 25083768 PMC4118852

